# A natural light-driven inward proton pump

**DOI:** 10.1038/ncomms13415

**Published:** 2016-11-17

**Authors:** Keiichi Inoue, Shota Ito, Yoshitaka Kato, Yurika Nomura, Mikihiro Shibata, Takayuki Uchihashi, Satoshi P. Tsunoda, Hideki Kandori

**Affiliations:** 1 Department of Life Science and Applied Chemistry, Nagoya Institute of Technology, Showa-ku, Nagoya 466-8555, Japan; 2 OptoBioTechnology Research Center, Nagoya Institute of Technology, Showa-ku, Nagoya 466-8555, Japan; 3 PRESTO, Japan Science and Technology Agency, 4-1-8 Honcho, Kawaguchi, Saitama 332-0012, Japan; 4 Department of Physics, Kanazawa University, Kanazawa 920-1192, Japan; 5 Bio-AFM Frontier Research Center, Kanazawa University, Kanazawa 920-1192, Japan

## Abstract

Light-driven outward H^+^ pumps are widely distributed in nature, converting sunlight energy into proton motive force. Here we report the characterization of an oppositely directed H^+^ pump with a similar architecture to outward pumps. A deep-ocean marine bacterium, *Parvularcula oceani*, contains three rhodopsins, one of which functions as a light-driven inward H^+^ pump when expressed in *Escherichia coli* and mouse neural cells. Detailed mechanistic analyses of the purified proteins reveal that small differences in the interactions established at the active centre determine the direction of primary H^+^ transfer. Outward H^+^ pumps establish strong electrostatic interactions between the primary H^+^ donor and the extracellular acceptor. In the inward H^+^ pump these electrostatic interactions are weaker, inducing a more relaxed chromophore structure that leads to the long-distance transfer of H^+^ to the cytoplasmic side. These results demonstrate an elaborate molecular design to control the direction of H^+^ transfers in proteins.

## Abstract (web summary)

Proton pumps that are driven by light to pump protons out of the cell are involved in the conversion of sunlight into proton motive force; pumps to drive protons in the other direction have been engineered. Here, the authors report the identification and characterisation of a naturally occurring inward-driven protein pump.

## Graphical abstract

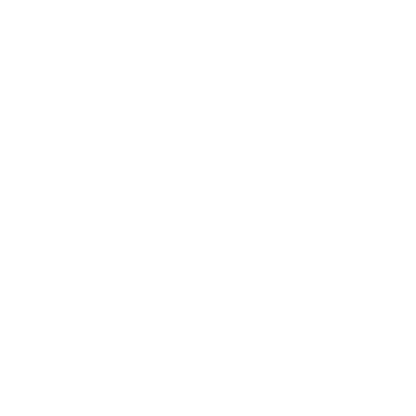

Microorganisms utilize ion-transporting rhodopsins such as light-driven pumps and light-gated channels for electrochemical membrane potential generation and signal transduction, respectively^1^. These rhodopsins are also important tools for optogenetics, which control neural activity by light^2^. While light-driven outward H^+^ and inward Cl^−^ pumps were discovered in the last century^3^,^4^,^5^, more recent metagenomic analyses led to the discovery of an outward Na^+^ pump^6^, and cation^7^,^8^ and anion channels^9^. Figure 1 summarizes the functions of ion-transporting rhodopsins in which transport is uni-directional for pumps and bi-directional for channels^1^. The direction of transport for known retinal-binding ion pumps is exclusively outward for cations and inward for anions, increasing membrane potential. The presence of inward cation and outward anion pumps is highly unlikely in nature, as it is energetically unfavourable.

Previously, we engineered inward H^+^ transport by mutating *Anabaena* sensory rhodopsin (ASR)^10^, a photochromic light sensor. Wild-type ASR does not transport ions, but an ASR mutant (D217E) exhibited light-induced inward H^+^ transport when expressed in *Escherichia coli* cells. D217 is located in the cytoplasmic region of ASR, and light-induced difference Fourier transform infrared (FTIR) spectroscopy clearly showed an increased proton affinity for E217, which presumably controls the unusual directionality opposite to that in normal proton pumps. However, in that paper, we could not determine if the mutant functioned as an H^+^ pump or channel as the inside of the cell was negatively charged. On a related note, conversions of light-driven outward H^+^ pumps into an H^+^ channel by mutation were reported recently^11^,^12^.

Here we report that a microbial rhodopsin from a deep-ocean marine bacterium, *Parvularcula oceani*, functions as inward H^+^ pump when expressed in *E. coli* and mouse neural cells. Mechanistic analyses of purified proteins reveal that the retinal chromophore structure and primary photoisomerization (C_13_=C_14_ *trans* to *cis*) are identical between outward and inward H^+^ pumps. Nevertheless, the direction of primary H^+^ transfer differs because of different electrostatic interactions of the protonated Schiff base with its counterion. We discuss these findings in terms of the molecular mechanisms of light-driven inward and outward H^+^ pumps.

## Results

### *Po*XeR is a light-driven inward H^+^ pump

**Figure 2:**
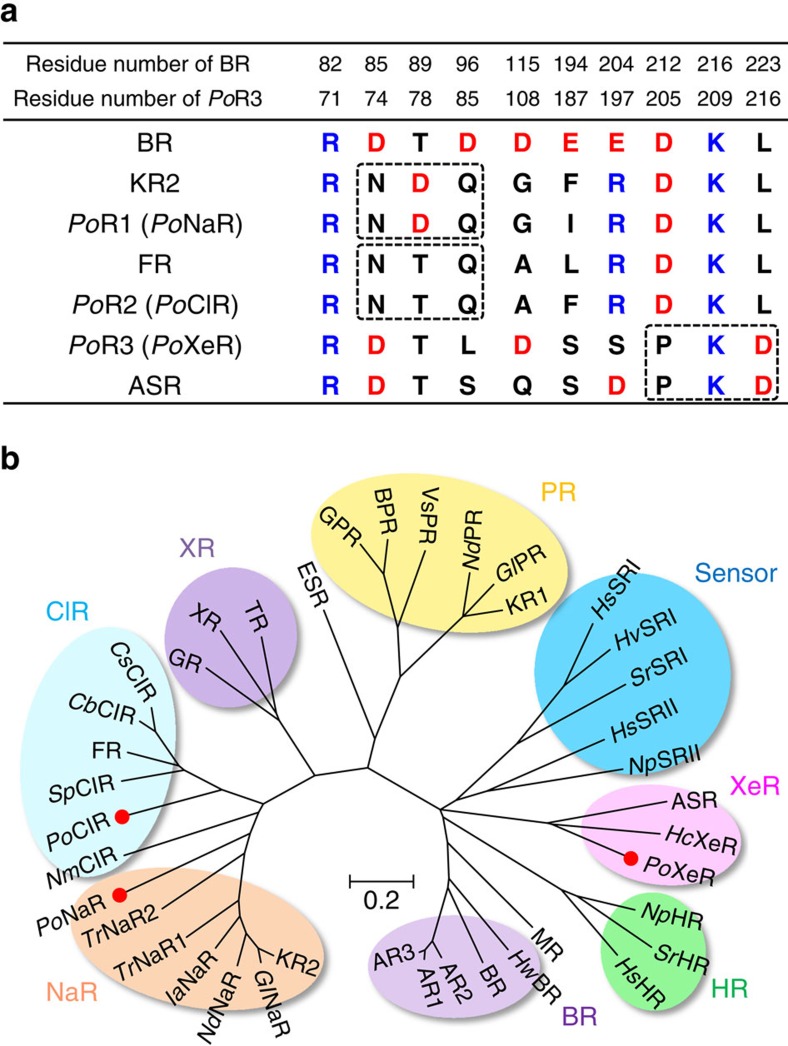
Amino-acid sequence alignment of important residues and phylogenetic tree of microbial rhodopsins. (**a**) Important residues for the function of microbial rhodopsins. BR, KR2, FR and ASR are outward H^+^ pump, outward Na^+^ pump, inward Cl^−^ pump, and photochromic sensor, respectively. Dashed rectangles indicate the positions of NDQ and NTQ motifs and residues unique for XeR. (**b**) Phylogenetic tree of selected microbial rhodopsins. The rhodopsins included in the phylogenetic tree: bacteriorhodopsin from *Halobacterium salinarum* (BR), archaerhodopsin-1, -2 and -3 from *Halorubrum sodomense* (AR1, AR2 and AR3), bacteriorhodopsin and middle rhodopsin from *Haloquadratum walsbyi* (*Hw*BR, MR), halorhodopsin from *H. salinarum*, *Salinibacter ruber* and *Natronomonas pharaoni*s (*Hs*HR, *Sr*HR, *Np*HR), *Po*XeR, xenorhodopsin from *Haloplasma contractile* (*Hc*XeR), ASR, sensory rhodopsin II from *N. pharaonis* and *H. salinarum* (*Np*SRII and *Hs*SRII), sensory rhodopsin I from *S. ruber*, *Haloarcula vallismortis* and *H. salinarum* (*Sr*SRI, *Hv*SRI and *Hs*SRI), proteorhodopsin from *Krokinobacter eikastus*, *Gillisia limnaea*, *Nonlabens dokdonensis* and *Vibrio sp. AND4* (KR1, *Gl*PR, *Nd*PR, *Vs*PR), blue-absorbing proteorhodopsin from uncultured bacterium (BPR), green-absorbing proteorhodopsin from uncultured marine gamma proteobacterium (GPR), rhodopsin from *Exiguobacterium sibiricum* (ESR), thermophilic rhodopsin from *Thermus thermophilus* (TR), xanthorhodopsin from *S. ruber* (XR), rhodopsin from *Gloeobacter violaceus PCC 7421* (GR), putative Cl^−^ pumping rhodopsin from *Citromicrobium sp. JLT1363*, *Citromicrobium bathyomarinum* and *Sphingopyxis baekryungensis* (*Cs*ClR, *Cb*ClR, *Sp*ClR), Cl^-^ pump from *Fulvimarina pelagi* and *Nonlabens marinus S1-08* (FR and *Nm*ClR), *Po*ClR, *Po*NaR, two putative NaRs from *Truepera radiovictrix* (*Tr*NaR1 and *Tr*NaR2), putative NaR from *Indibacter alkaliphilus* (*Ia*NaR), NaR from *N. dokdonensis*, *G. limnaea* and *K. eikastus* (*Nd*NaR, *Gl*NaR and KR2).

*P. oceani* is an α-proteobacterium found at a depth of 800 m in the south-eastern Pacific ocean. Analysis of the genome of *P. oceani* showed the existence of three microbial rhodopsins^13^. Two rhodopsins contain the NDQ and NTQ motifs, suggesting light-driven outward Na^+^ (*Po*NaR) and inward Cl^−^ (*Po*ClR) transport, respectively (Fig. 2). The remaining rhodopsin possesses the DTL motif (Fig. 2a) and has amino-acid sequence 51% identical to that of ASR^14^, a photochromic light sensor (Supplementary Fig. 1). Nevertheless, unlike ASR, this rhodopsin does not contain a long Arg-rich C-terminus (Supplementary Fig. 1), and can be classified as a xenorhodopsin (XeR), whose function is unknown^15^ (Supplementary Fig. 1). Thus, unlike many microbes that normally contain an H^+^ pump, *P. oceani* does not seem to have one. Instead, it has an Na^+^ pump (*Po*NaR), a Cl^−^ pump (*Po*ClR) and rhodopsin of an unidentified function (*Po*XeR).

**Figure 3:**
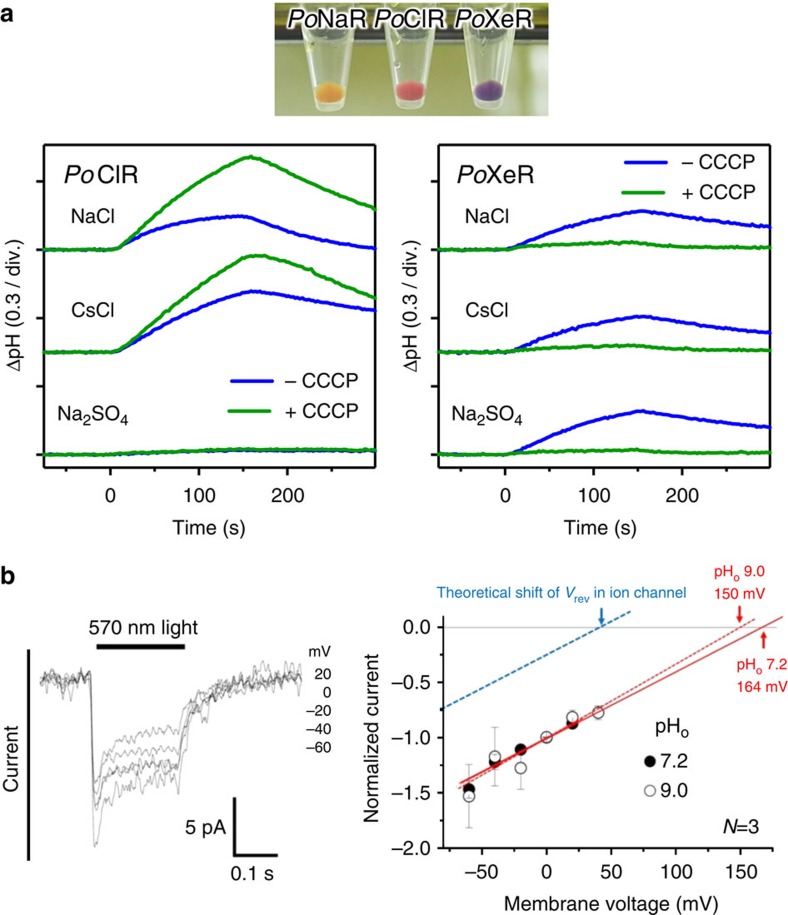
Light-driven H^+^ transport activity of *Po*XeR. (**a**) *E. coli* C43(DE3) strain cells in which the expression of *Po*NaR, *Po*ClR and *Po*XeR was induced (upper), and ion-pumping activity was assayed by observing pH changes (lower). Light is on between 0 and 150 s. (**b**) Electrophysiological measurements of *Po*XeR-driven photocurrent in ND7/23 cells (left) and *I*–*V* plot of the current at pH_o_ 7.2 and 9.0.

We tested the ion-transporting functions of these rhodopsins using heterologous expression of C-terminally his-tagged proteins in *E. coli*. The membranes of *Po*NaR-expressing bacteria were yellow (Fig. 3a), suggesting low expression or improper folding of the protein. In contrast, *Po*ClR and *Po*XeR formed red/purple pigment (Fig. 3a). Next, we examined their ion-pumping activities. For *Po*ClR-expressing cells, we observed a light-induced increase in pH, which was accelerated by carbonylcyanide-m-chlorophenylhydrazone (CCCP) (Fig. 3a). This pH increase was similar in NaCl and CsCl, but was abolished when Na_2_SO_4_ was used. Strong anion dependence is fully consistent with the inward Cl^−^ pump function of *Po*ClR. For *Po*XeR-expressing cells, we also observed a light-induced pH increase in all salts, but the signals disappeared in the presence of CCCP (Fig. 3a). This suggests inward H^+^ transport driven by light for *Po*XeR. Even though ASR does not transport ions^14^, we engineered inward H^+^ transport of an ASR mutant (D217E) by light^10^. However, it was not clear D217E ASR is an inward H^+^ pump or channel, because the inside of the cell is negatively charged. To verify the active nature of inward proton transport, voltage and current across the membrane should be controlled. For this purpose, we expressed *Po*XeR in mouse ND7/23 cells and performed electrophysiological measurements. The data in Fig. 3b display an inward current for all measured membrane voltages (from −55 mV to 45 mV). More importantly, the obtained *I*–*V* curve was identical for different extracellular pH values (7.2 and 9.0), typical for light-driven H^+^ pumps. Thus, despite the lack of measurements in native cells, the results in *E. coli* and ND7/23 cells strongly suggest that *Po*XeR is a natural light-driven inward H^+^ pump.

### Molecular properties of *Po*XeR

The unusual inward H^+^ pumping activity of *Po*XeR poses questions about its physiological role and molecular mechanism. The former is difficult to address as the culture of native cells is not available. As for the latter, we studied the molecular mechanism of the inward H^+^ pumping by various spectroscopic methods using *Po*XeR expressed in *E. coli*.

**Figure 4:**
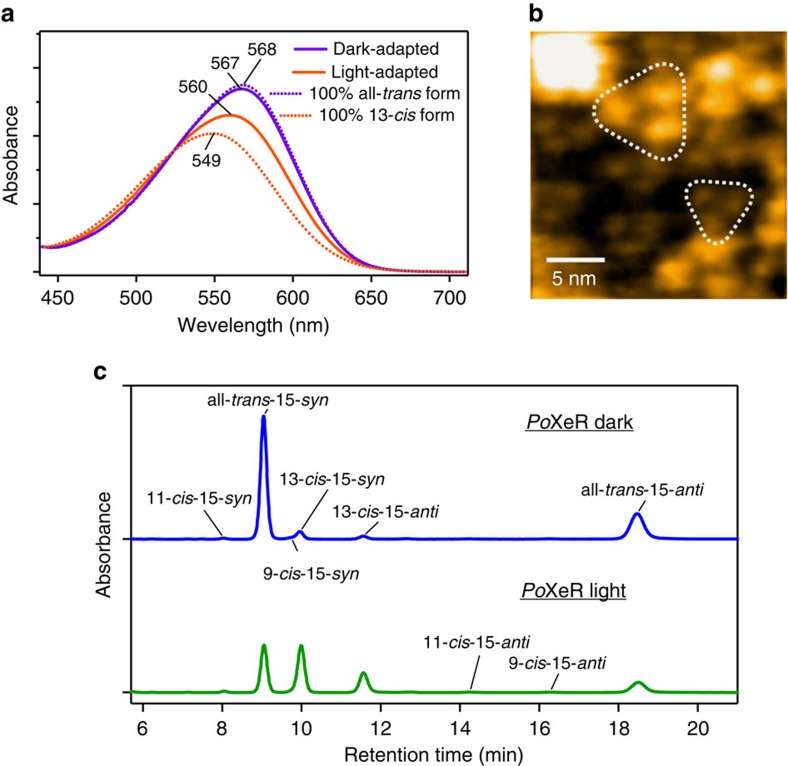
Molecular properties of *Po*XeR. (**a**) Absorption spectra of dark- and light-adapted *Po*XeR (purple and orange solid lines, respectively). Dashed lines represent the calculated spectra of 100% all-*trans* and 100% 13-*cis* forms. (**b**) High-speed AFM image of *Po*XeR reconstituted in a nanodisc. (**c**) HPLC pattern of retinal extracted from *Po*XeR.

Figure 4a shows the absorption spectrum of *Po*XeR (*λ*_max_=567 nm) in the dark and after illumination, and a high-speed atomic force microscopy (AFM) image (Fig. 4b) shows that *Po*XeR forms a trimer in the nanodisk membrane. ASR contains predominantly all-*trans* chromophore in the dark but the 13-*cis*, 15-*syn* chromophore is formed after light-adaptation^16^,^17^. This is also the case for *Po*XeR, which is 92% all-*trans* in the dark while all-*trans* and 13-*cis* forms are equally distributed after the illumination (Fig. 4c). Calculated absorption spectra of all-*trans* and 13-*cis* forms exhibit *λ*_max_ at 568 and 549 nm, respectively, similar to those observed for ASR^18^.

### Photoreaction dynamics of a dark-adapted *Po*XeR

**Figure 5:**
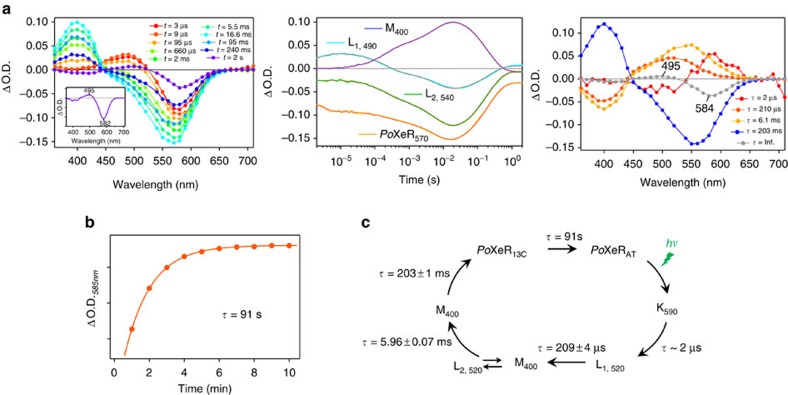
Photoreaction dynamics of *Po*XeR. (**a**) Transient difference absorption spectra of dark-adapted *Po*XeR (left) and absorption difference time-evolution at specific wavelengths (centre). Decay-associated spectra obtained by global fitting of the changes in transient absorption (right). The inset shows the difference spectrum between light-adapted and dark-adapted state. (**b**) The process of dark-adaptation estimated by the recovery of absorption at 585 nm. (**c**) The photocycle of *Po*XeR. Values of the time-constants of each process are the mean±s.d.

We next studied the photocycle of the dark-adapted *Po*XeR protein, which presumably represents the inward H^+^ pumping process. To avoid photoexcitation of the 13-*cis* form, we measured single-wavelength kinetics of a 0.6 ml sample of dark-adapted protein, and replaced it after each single excitation measurement. Figure 5a shows the time-resolved difference spectra (left), absorption changes at each wavelength (centre) and decay-associated spectra (right). Upon photoexcitation of the all-*trans* form (*Po*XeR_AT_), the red-shifted K intermediate appears first, and decays to the blue-shifted L intermediate in 2 μs. Then, the M intermediate containing a deprotonated Schiff base appears with two distinct time-constants (210 μs and 6.0 ms). This analysis shows the presence of two states for L and M, one of which is in equilibrium. The M-intermediate decays in 200 ms, but the transient absorption does not return to zero (Fig. 5a, centre) at >1 s, indicating the presence of another long-lived intermediate state. The difference spectrum obtained by transient absorption spectroscopy (spectrum at 2 s in Fig. 5a, left) coincides with that obtained by steady-state absorption measurements in Fig. 4a (Fig. 5a, inset). Therefore, the last intermediate represents metastable 13-*cis* state (*Po*XeR_13C_) that reverts into *Po*XeR_AT_ in 91 s (Fig. 5b). The photocycle of *Po*XeR_AT_ is summarized in Fig. 5c: the inward H^+^ transport is made possible by (i) H^+^ release from the Schiff base to the cytoplasmic region upon M formation and (ii) H^+^ uptake from the extracellular region upon M decay.

### H^+^ pathway during the inward transport in *Po*XeR

**Figure 6:**
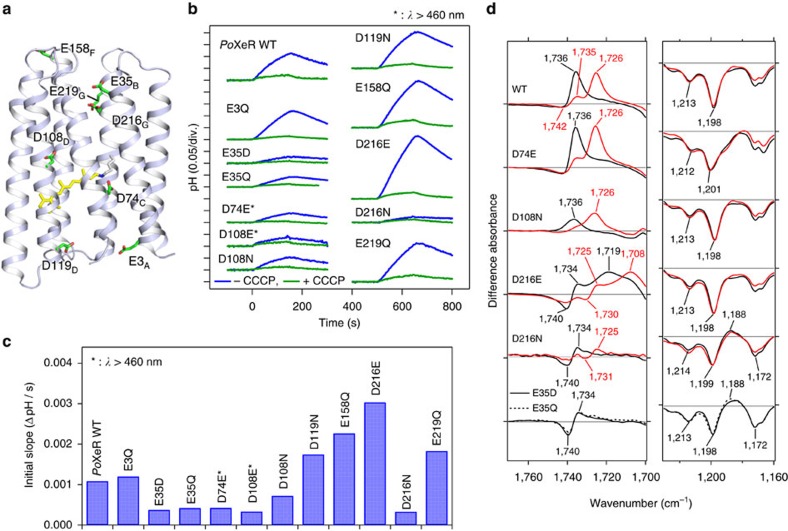
Inward H^+^ pumping activity and light-induced FTIR spectra of *Po*XeR and its mutants. (**a**) The crystal structure of ASR (PDB ID: 1XIO)^21^ is shown with the side chains of the acidic residues used in *Po*XeR mutagenesis studies. The residue numbering of PoXeR is shown. (**b**) H^+^ transport activity of *Po*XeR mutants in *E. coli* cells after normalization for the amount of protein. Light is on between 0 and 150 s. (**c**) The initial slopes of light-induced pH changes shown in **b**. (**d**) Light-induced difference FTIR spectra of WT *Po*XeR and the mutants at *T*=230 K and pH 8.0. Spectra are measured in H_2_O (black) and D_2_O (red).

As light-driven outward H^+^ pumps contain internal H^+^ acceptors and donors^1^, we examined candidates for such groups for the inward H^+^ pump. Membrane-embedded carboxylates are strong candidates for internal H^+^ acceptors and donors, as is the case for D85 (acceptor) and D96 (donor) in an outward H^+^ pump BR (refs 19, 20). Figure 6a shows putative location of eight intramembrane carboxylates possibly involved in the transport, which we replaced with neutral residues (D-to-N or E-to-Q). Among the mutants, only D74N showed the absence of colour. As D74 acts as the counterion of the protonated Schiff base, we attempted a more conservative replacement, D74E. The inward H^+^ pumping activity was measured for various mutants (Fig. 6b), and the initial slopes are plotted in Fig. 6c.

The results clearly show a significant reduction in the pumping activity in E35D/Q, D74E, D108E/N and D216N. In contrast, D216E showed about threefold higher inward H^+^ pumping activity than wildtype (WT). It should be noted that the corresponding mutant of ASR (D217E) shows inward H^+^ transport^10^. Previously, light-induced difference FTIR spectroscopy of D217E ASR revealed protonation of E217 upon deprotonation of the Schiff base (M intermediate)^10^. Here we applied similar FTIR analysis to *Po*XeR_AT_ (Supplementary Fig. 2). The top panel of Fig. 6d shows a positive peak at 1,736 cm^−1^, which down-shifts to 1,726 cm^−1^ in D_2_O in the M-minus-*Po*XeR_AT_ difference spectra. This is a characteristic signal of protonated carboxylic acids, indicating that carboxylate is the H^+^ acceptor of the Schiff base. Similar positive peaks were observed for D74E and D108N, but an entirely different spectral feature was obtained for D216E. Sharp positive peaks of WT at 1,736 and 1,726 cm^−1^ in H_2_O and D_2_O were shifted to 1,719 and 1,708 cm^−1^ in D216E, respectively, and the remaining pair of peaks at 1740 (−)/1734 (+) cm^−1^ in H_2_O (1,730 (−)/1,725 (+) cm^−1^ in D_2_O) originates from a different carboxylic acid. This strongly suggests that D216 is the H^+^ acceptor during the inward H^+^ transport in *Po*XeR. In agreement with that idea, the positive peak at 1,736 cm^−1^ was absent in D216N. In contrast, a positive peak was observed at 1,188 cm^−1^ in the C–C stretching region of retinal chromophore, indicating that the M intermediate did not form in D216N. There was no positive peak in the frequency region of protonated carboxylic acids and the 1,188 cm^−1^ band was present (no M formation) in E35Q/D as well. From homology modelling, E35 is likely to be located near D216, and the E35 mutation may raise the pKa of D216, leading to the lack of M formation. According to the structure of ASR, the distance between the Schiff base and D217 (D216 in *Po*XeR) is 14.7 Å (ref. 21), and such long-range H^+^ transfer should be mediated by other residues and/or water molecules in the inward H^+^ pump.

**Figure 7:**
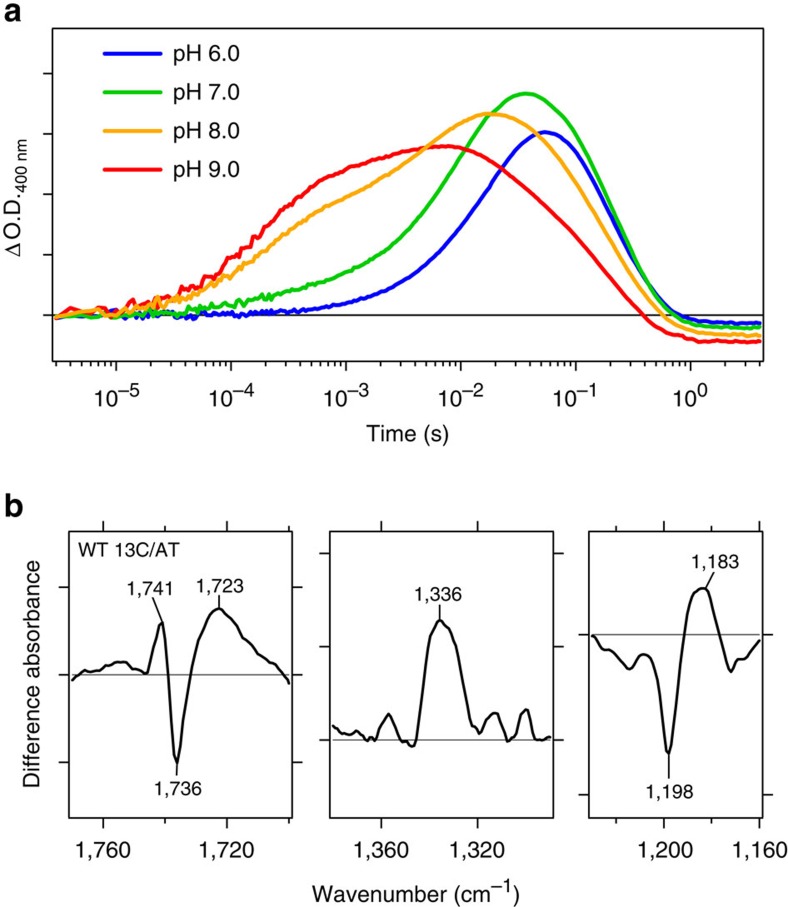
Analysis of H^+^ uptake in *Po*XeR. (**a**) Time-evolution of the accumulation of M intermediate at various pH values. (**b**) Light-induced difference FTIR spectra between *Po*XeR_13C_ and *Po*XeR_AT_ states at 277 K and pH 8.0.

As for the identity of H^+^ donor to the Schiff base, the decay of the M intermediate accompanies protonation of the Schiff base, and if H^+^ is taken up from the aqueous phase, the decay of M should slow down at high pH (ref. 22). We thus measured the M decay kinetics at different pH values. Figure 7a shows strong pH dependence of the M rise but limited pH dependence of the M decay. In fact, the speed of M decay increased at pH 9.0. This fact indicates the presence of an internal H^+^ donor. If it is a carboxylic acid, we expect a negative band in the 1,760–1,700 cm^−1^ region in the *Po*XeR_13C_-minus-*Po*XeR_AT_ difference FTIR spectra. However, Fig. 7b shows a broad positive peak at 1,723 cm^−1^ in addition to a peak pair at 1,741 (+)/1,736 (−) cm^−1^. Absence of deprotonation signal in this region questions the role of a carboxylic acid as the H^+^ donor. Other residues such as arginine and protein-bound water molecules are possible candidates for the internal H^+^ donor^23^,^24^. The spectral features at 1,336 (+) cm^−1^ and 1,198 (−)/1,183 (+) cm^−1^ (Fig. 7b and Supplementary Fig. 3) resemble those of the dark adaptation in BR^25^, suggesting that *Po*XeR_13C_ contains the 13-*cis*, 15-*syn* configuration.

## Discussion

**Figure 8:**
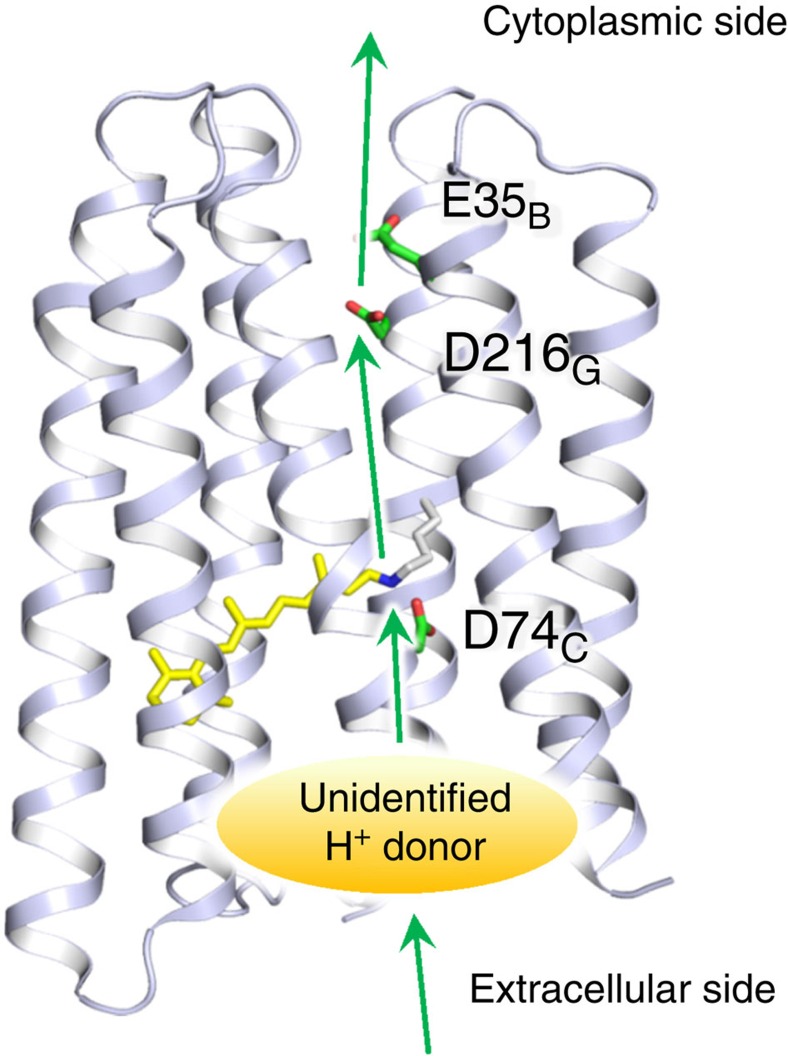
Model for H^+^ transfer pathway in *Po*XeR. The H^+^ transfer pathway in *Po*XeR suggested by the results of the present study. The primary H^+^ transfer occurs from the Schiff base to D216, and E35 affects this reaction. The Schiff base is not directly reprotonated from the aqueous phase, but there is an internal H^+^ donor, yet to be identified.

In this paper, we report the discovery and characterization of a natural retinal-binding inward H^+^ pump (*Po*XeR). Mechanistic analyses of the purified protein revealed that the retinal chromophore structure and primary photoisomerization (C_13_=C_14_ *trans* to *cis*) are identical between outward and inward H^+^ pumps. Nevertheless, direction of the primary H^+^ transfer in *Po*XeR is opposite to that in outward H^+^ pumps. The pathway of the inward H^+^ transport in *Po*XeR is summarized in Fig. 8. The primary H^+^ transfer occurs from the Schiff base to D216 on the cytoplasmic side. E35 modulates its pKa by forming hydrogen-bonding network with D216, similar to the one reported for ASR (E36 and D217)^26^. Secondary H^+^ transfer occurs from an unidentified group to the Schiff base on the extracellular side. This sequence of events in H^+^ transport is entirely opposite to the one well-known for outward H^+^ pumps such as BR^1^,^27^,^28^,^29^,^30^,^31^,^32^,^33^.

**Figure 9:**
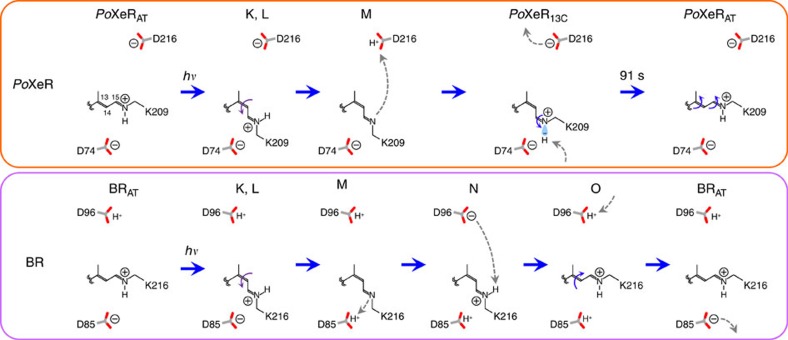
Mechanism of inward H^+^ transport in *Po*XeR and outward H^+^ transport in BR. Schematic description of H^+^ transport in the photocycle of *Po*XeR compared with the photocycle of BR. In BR, only one double bond at C_13_=C_14_ isomerizes during the outward H^+^ pumping photocycle (*trans* to *cis* photoisomerization, and *cis* to *trans* thermal isomerization). In contrast, retinal isomerization is more complex to facilitate the inward H^+^ pump in *Po*XeR: (i) C_13_=C_14_ *trans* to *cis* photoisomerization, (ii) C_15_=N *anti* to *syn* thermal isomerization, and (iii) C_13_=C_14_ *cis* to *trans* and C_15_=N *syn* to *anti* thermal isomerization by a bicycle-pedal motion.

We suggest the following mechanism for the distinct vectoriality between *Po*XeR and BR transport (Fig. 9). Photoexcitation first converts the all-*trans* chromophore into a twisted 13-*cis*, 15-*anti* state in both, but its relaxation differs between BR and *Po*XeR. There are two negative charges, D85 and D212, on the extracellular side of BR, producing strong electrostatic attraction of the protonated Schiff base before the primary H^+^ transfer, leading to outward H^+^ transport^34^. In contrast, the XeR family possesses only one negative charge in that region, as D212 of BR is replaced by proline, which makes the electrostatic interaction between the protonated Schiff base and the counterion (D74 in *Po*XeR) weaker^35^,^36^,^37^,^38^. Consequently, photoisomerization reorients the N–H group of the Schiff base toward the cytoplasmic side. The cytoplasmic domain is more polar in ASR than in BR^21^, and such a structure should promote long-distance H^+^ transfer to D216 in *Po*XeR.

After the M-intermediate formation, BR receives H^+^ from the cytoplasmic side, followed by thermal isomerization to the original all-*trans* form. Therefore, only one double bond at C_13_=C_14_ bond isomerizes during the functional photocycle of BR. In contrast, after M formation of *Po*XeR, it is likely that thermal isomerization occurs at C_15_=N bond from an *anti* to a *syn* form, followed by secondary H^+^ transfer on the extracellular side. This yields a 13-*cis*, 15-*syn* form (*Po*XeR_13C_), and thermal bicycle-pedal isomerization^39^ (13-*cis*, 15-*syn* to all-*trans*, 15-*anti*) reverts it to the original state. Thermal isomerization of the C_15_=N group was known for BR as a dark-adaptation process^25^, but is directly related to the function of XeRs, such as ASR and *Po*XeR. In ASR, C_15_=N thermal isomerization after C_13_=C_14_ photoisomerization occurs with 100% yield, leading to an efficient photochromic reaction^18^,^40^. In *Po*XeR, C_15_=N thermal isomerization allows for an inward H^+^ transport, an additional function for microbial rhodopsins, though the thermal isomerization yield is unknown at present.

Inward H^+^ transport may lower the proton motive force, which is bioenergetically disadvantageous for marine bacteria. Thus, its presence in nature suggests a different physiological role, not related to bioenergetics. One possibility is that the inward H^+^ pump may be used for intracellular signal transduction, similar to ASR^14^. A future structure–function study of *Po*XeR should lead to a better understanding of this unique function. The light-driven inward H^+^ pump is also a potential tool for optogenetics. The light-driven outward H^+^ pump Arch has been used as a neural silencer in optogenetics and Arch is also used for acidification of cell organelles^41^. An oppositely directed light-driven H^+^ pump would further enable the control of cell organelles.

In summary, we show that *Po*XeR is a light-driven inward H^+^ pump from a marine α-proteobacterium. While outward H^+^ pumps are widely present in nature, oppositely directed H^+^ pumps are rare. In inward H^+^ pumps, the lack of a negative charge in the active centre causes weak coupling to the Schiff base counterion (D74) in the key photocycle intermediate, leading to the release of H^+^ to D216 in the cytoplasmic region. Retinal isomerization sequence became more complex to facilitate the inward H^+^ transport: (i) C_13_=C_14_ *trans* to *cis* photoisomerization, (ii) C_15_=N *anti* to *syn* thermal isomerization and (iii) C_13_=C_14_ *cis* to *trans* and C_15_=N *syn* to *anti* thermal isomerization by a bicycle-pedal motion^39^. This work demonstrates an elaborate molecular design to control the direction of H^+^ transport in retinal-binding proteins. The discovery of a light-driven inward H^+^ pump will lead to better understanding of the molecular mechanism of light-driven pumps and contribute to the development of new optogenetic tools.

## Methods

### Protein expression and purification

Genes of *Po*XeR, *Po*NaR and *Po*ClR, whose codons were optimized for an *E. coli* expression system, were synthesized (Eurofins Genomics Inc.) and subcloned into a pET21a(+)-vector with C-terminal 6 × His-tag. For mutagenesis, a QuikChange site directed mutagenesis kit (Stratagene) was used according to a standard protocol. Wildtype and mutant proteins were expressed in *E. coli* C43(DE3) strain. Protein expression was induced by 0.21 mM isopropyl β-D-thiogalactopyranoside (IPTG) for 4 h at 37 °C when 10 μM all-*trans*-retinal (Sigma-Aldrich) was supplemented in the culture. The expressed proteins were purified from *E. coli* cells using previously reported protocols^6^,^42^,^43^,^44^. The cells were disrupted by a French Press (Ohtake) and the membrane fraction was collected by ultracentrifugation (125,000*g*, 1 h). The protein was solubilized with 2% *n*-dodecyl-β-D-maltoside (DDM) (Anatrace) in the presence of 300 mM NaCl, 5 mM imidazole and 50 mM MES (pH 6.5). After Co^2+^-NTA affinity chromatography, the collected fractions were dialyzed against a solution containing 50 mM Tris–HCl pH 8.0, 100 mM NaCl, 0.1% DDM to remove imidazole used for the elution from a column.

### Assay of light-driven ion-pumping activity of rhodopsins

*E. coli* cells expressing rhodopsins were collected by centrifugation (4,800*g*, 3 min), washed three times with and resuspended in aqueous solution containing 100 mM salt (NaCl, CsCl and Na_2_SO_4_). Cell suspension of 7.5 ml at OD_660_=2 was placed in the dark and then illuminated at *λ*>500 nm by a 1-kW tungsten–halogen projector lamp (Rikagaku, Japan) through a glass filter (Y-52, AGC Techno Glass, Japan). For the blue-shifted *Po*XeR mutants (*Po*XeR D74E and D108E), light of *λ*>460 nm (Y-48, AGC Techno Glass, Japan) was used for the photoexcitation. The light-induced pH changes were measured by a pH electrode (HORIBA, Japan). Measurements were repeated under the same conditions after the addition of 10 μM CCCP.

### Quantification of rhodopsins expressed in *E. coli*

The amount of rhodopsin expressed in *E. coli* was estimated by a previously reported method^6^,^43^,^44^. *E. coli* cells expressing rhodopsins were collected by centrifugation at 3,600*g* and 4 °C and suspended in a solution containing 100 mM NaCl and 50 mM Tris–HCl (pH 8.0), to a final volume of 3 ml. Then, 200 μl of 1 mM lysozyme was added to the suspension and it was gently stirred at room temperature for 1 h. The *E. coli* cells were disrupted by sonication (TAITEC, Japan) and solubilized in 3.0% DDM. The change in absorption, which represents the bleaching of rhodopsin by hydroxylamine (HA), was measured with a ultraviolet–vis spectrometer (Shimadzu, Japan) equipped with an integrating sphere after the addition of HA to a final concentration of 500 mM and illumination at *λ*>500 nm by a 1-kW tungsten–halogen projector lamp (Rikagaku, Japan) through a glass filter (Y-52, AGC Techno Glass, Japan). The molecular extinction coefficient of WT *Po*XeR and mutant proteins (*ɛ*) was calculated from the ratio between the absorbance of rhodopsin and retinal oxime (*ɛ*=33,600 M^−1^ cm^−1^ at 360 nm (ref. 45) produced by the reaction between the retinal Schiff base and HA. The amount of protein expressed in *E. coli* cells was determined by the absorbance of the bleached rhodopsin. The transport activity of *E. coli* cells containing each rhodopsin was quantitatively determined from the initial slope of pH change after normalizing by the expression level of protein.

### Measurement of dark-adaptation kinetics

Dark adaptation kinetics was measured for 0.1% DDM-solubilized sample at 24 °C. The dark-adapted sample was illuminated for 2 min by using output from a 1-kW tungsten–halogen projector lamp (Master HILUX-HR, Rikagaku, Japan) through a glass filter (O-60, AGC Techno Glass, Japan) at *λ*>580 nm. After turning off the light, the spectra or the absorption at a specific wavelength were measured every 1 min or 1 s, respectively, by a ultraviolet–visible spectrometer (V-730, JASCO).

### Heterologous expression in mammalian cells

A human codon-adapted *Po*XeR gene was synthesized by Gen Script (Piscataway, NJ, USA) and cloned into pEGFP vector between *Hind*III and *Bam*HI sites. ND7/23 cells were purchased from DS Pharma Biomedical (Osaka, Japan) and cultured in high-glucose DMEM media (Wako) in a 37 °C, 5% CO_2_ incubator. Transfection of ND7/23 cells was performed by Lipofectamine 2000 (Invitrogen, Carlsbad, CA, USA). Cells were supplemented with 1 μM all-*trans*-retinal (Sigma-Aldrich) after transfection. Expression was confirmed by a fluorescence microscope (IX-73, Olympus, Tokyo, Japan).

### Electrophysiology

Whole cell patch clamp was performed with an amplifier, Axopatch 200B (Molecular Devices, Sunnyvale, CA, USA). Continuous light was provided by OSG L12194-00-39070 (Hamamatsu Photonics, Shizuoka, Japan) via a light guide into an inverted microscope, IMT-2 (Olympus, Tokyo Japan). Illumination was controlled by a mechanical shutter LS6S (Vincent Associates, Rochester, NY, USA). Glass pipettes were fabricated by a micropipette puller, P-97 (Sutter Instrument, Novato, CA, USA) and fire-polished by a micro forge, MF-830 (Narishige, Tokyo, Japan). The pipette resistance was 1.5–2.5 MΩ. The pipette electrode was controlled by a micro manipulator, PCS-5000 (Burleigh instruments, Fishers, NY, USA). Current traces were recorded at 10 kHz and filtered to 2 kHz by an internal circuit of the amplifier. Data acquisition and shutter triggering were performed by pClamp 10 software via a Digidata 1550 (Molecular Devices, Sunnyvale, CA, USA). Data were analysed by Clampfit 10 software.

The standard external solution contained 140 mM NaCl, 2 mM MgCl_2_, 2 mM CaCl_2_, 2 mM KCl and 10 mM HEPES-NaOH (pH 7.2). The standard internal solution contained 110 mM NaCl, 2 mM MgCl_2_, 1 mM CaCl_2_, 5 mM KCl, 10 mM EGTA and 10 mM HEPES-NaOH (pH 7.2). Osmolality of the solutions was adjusted to 300 mOsm by adding an appropriate amount of sucrose.

### Preparation of *Po*XeR reconstituted in asolectin

For *Po*XeR reconstitutions in a lipid bilayer, we used a nanodisc protocol^46^,^47^,^48^. Briefly, *Po*XeR (∼5 nM) solubilized in 0.1% DDM, membrane scaffold proteins (MSP1E3D1) (M7074, Sigma) (∼30 μM) and asolectin (11145, Sigma) (∼120 μg) were mixed in a solution containing 20 mM HEPES (pH 7.4) and 100 mM NaCl. The mixture was incubated for 1 h at 4 °C and then Bio-Beads SM-2 (Bio-Rad) (60 mg) were added to remove the detergent. After mixing overnight at 4 °C, Bio-Beads were removed by centrifugation at 14,000 r.p.m. and 4 °C for 1 min. The nanodisc products were purified by a 0.45 μm syringe filter (Millipore).

### High-speed AFM

In-house high-speed AFM operated in tapping mode was used^49^,^50^. In high-speed AFM, the cantilever deflection is detected by an optical beam deflection detector using an infrared laser (780 nm). The laser beam is focused onto the back side of a cantilever covered by a gold film through a × 60 objective lens (Nikon: CFI S Plan Fluor ELWD 60 × ) and the reflected laser is detected by a two-segmented PIN photodiode. The size of a cantilever (Olympus: BL-AC7DS-KU4) is 6–7 μm long, 2 μm wide, and 90 nm thick. A spring constant is estimated to be 0.1–0.2 N m^−1^ by a thermal method, and a resonant frequency and quality factor of a cantilever in liquid are ∼1 MHz and ∼2, respectively. The free oscillation amplitude was ∼1 nm and the set-point amplitude was about 90% of the free amplitude during the AFM observation. To gain a sharp probe, we deposited an amorphous carbon tip on the initial bird's beak tip by electron beam deposition. The length of an amorphous carbon tip was ∼500 nm, and the end radius of the tip was ∼4 nm. The AFM was performed under the buffer solution containing 20 mM Tris–HCl (pH 8.0) and 100 mM NaCl at room temperature.

### HPLC analysis of retinal configuration

The configuration of retinal in *Po*XeR was analysed by high-performance liquid chromatograph (HPLC) as described previously^17^. A silica column (6.0 × 150 mm; YMC-Pack SIL) was used for the analysis. We added HA (final conc. 500 mM) to 100 μl *Po*XeR solution (100 mM NaCl, 20 mM Tris–HCl (pH 8.0)) at 4 °C, and then the protein was denatured with 66% (v/v) methanol. The retinal of *Po*XeR was released as retinal-oxime and extracted with hexane. The extracted sample was analysed by HPLC (solvent composition: 12% (v/v) ethyl acetate and 0.12% (v/v) ethanol in hexane) with 1.0 ml min^−1^ flow rate. The molar fraction of each retinal isomer was calculated from the ratio of the areas of corresponding peaks in the HPLC patterns. Each peak was assigned by comparison with the HPLC pattern of retinal oximes obtained from pure free all-*trans*- and 13-*cis* retinals. To analyse the retinal configuration of light-adapted *Po*XeR, the sample solution was illuminated with *λ*>500 nm light (Y-52, AGC Techno Glass) for 1 min before the reaction with HA at 4 °C. To estimate the experimental error, three identical measurements were performed for both dark- and light-adapted samples.

### Laser flash photolysis

The time-evolution of the transient absorption changes of photo-excited *Po*XeR was observed as previously described^6^. The purified sample was resuspended in buffer containing 50 mM Tris–HCl (pH 8.0), 100 mM NaCl and 0.1% DDM. The sample solution was placed in a quartz cuvette and was excited with a beam of second harmonics of a nanosecond pulsed Nd^3+^:YAG laser (*λ*=532 nm, INDI40, Spectra-Physics). The excitation laser power was 3 mJ pulse^−1^. Sample solution of 0.6 ml was used for each measurement and was replaced by a fresh dark-adapted sample for every photoexcitation. The change in transient absorption after photoexcitation was obtained by observing the change of the intensity of monochromated output of a Xe arc lamp (L9289-01, Hamamatsu Photonics, Japan) passed through the sample by a photomultiplier tube (R10699, Hamamatsu Photonics, Japan). Transient absorption spectra were reconstructed from the time-evolution of the change in transient absorption at various wavelengths from 360 to 710 nm with 10-nm intervals. The signals were global-fitted with a multi-exponential function and decay-associated spectra were obtained by plotting the pre-exponential factors against probed wavelengths.

### Low-temperature difference FTIR spectroscopy

For FTIR spectroscopy, WT *Po*XeR and mutants were reconstituted into a mixture of POPE and POPG (molar ratio=3:1) with a protein-to-lipid molar ratio of 1:50 by removing DDM with Bio-Beads (SM-2, Bio-Rad). The reconstituted samples were washed three times with 1 mM NaCl and 2 mM Tris–HCl (pH 8.0). The pellet was resuspended in the same buffer, but the concentration was adjusted to 1.7 mg ml^−1^. A 60 μl aliquot was placed onto a BaF_2_ window and dried with an aspirator. Low-temperature FTIR spectroscopy was applied to the films hydrated with H_2_O and D_2_O at 230 and 277 K as described previously^10^,^51^,^52^. For the formation of the M intermediate, samples were illuminated with >500 nm light for 1 s. To measure the difference infrared spectrum between *Po*XeR_AT_ and *Po*XeR_13C_, the latter was accumulated with >590-nm light for 1 min and spectra were measured during illumination. For each measurement, 128 interferograms were accumulated, and 2–4 identical recordings were averaged.

### Phylogenic analysis of rhodopsin genes

The amino-acid sequences of rhodopsins were aligned using MUSCLE programme^53^ after the removal of a weakly conserved interhelical loop, and N- and C-terminal extensions to increase the accuracy of alignment. The evolutionary history was inferred using the Neighbor-Joining method^54^. The optimal phylogenetic tree with the sum of branch length=15.12577349 was obtained. The percentage of replicate trees in which the associated taxa clustered together in the bootstrap test (1,000 replicates) were calculated^54^. The tree is drawn to scale, with branch lengths in the same units as those of the evolutionary distances used to infer the phylogenetic tree. The evolutionary distances were computed using the Poisson correction method^55^ and the units are the number of amino-acid substitutions/site. The analysis involved 41 amino-acid sequences. All positions containing gaps and missing data were eliminated. There were a total of 126 positions in the final dataset. Evolutionary analyses were conducted in MEGA6 (ref. 56).

### Data availability

The data that support the findings of this study are available from the corresponding author upon reasonable request. The structures of BR (PDB ID: 1M0L) and ASR (PDB ID: 1XIO) were used to highlight residues in transmembrane helices in Supplementary Fig. 1 and for illustrations purposes in Fig. 6 and Fig. 8.

## Additional information

**How to cite this article:** Inoue, K. *et al*. A natural light-driven inward proton pump. *Nat. Commun.* **7,** 13415 doi: 10.1038/ncomms13415 (2016).

**Publisher's note:** Springer Nature remains neutral with regard to jurisdictional claims in published maps and institutional affiliations.

## Supplementary Material

Supplementary Information Supplementary Figures 1-3

## Figures and Tables

**Figure 1:**
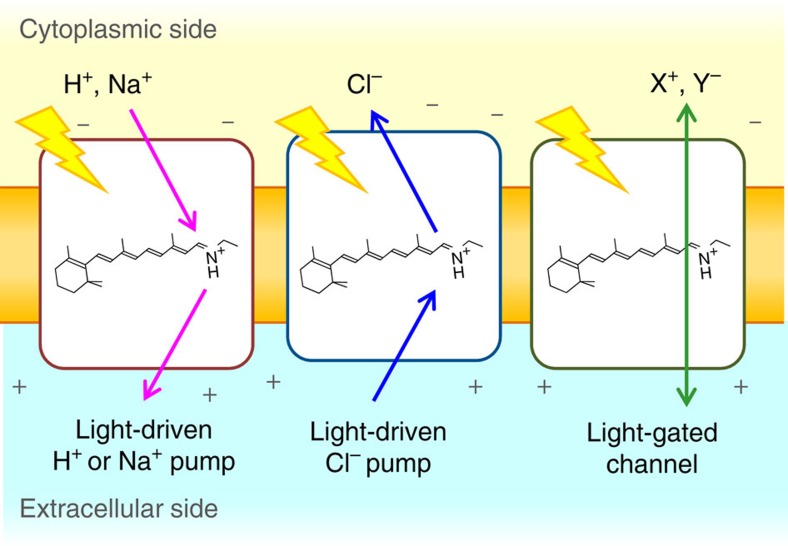
Ion-transporting microbial rhodopsins. Light-driven outward cation pumps (left) and inward anion pumps (middle) function as active transporters, while light-gated channels conduct cations or anions in a passive manner (right).
