# Supplementary material for: A natural light-driven inward proton pump
Source: Nat Commun. 2016 Nov 17;7:13415. doi: 10.1038/ncomms13415 (PMC5118547; doi:10.1038/ncomms13415)
Supplement: Supplementary Information — Supplementary Figures 1-3 [file ncomms13415-s1.pdf]

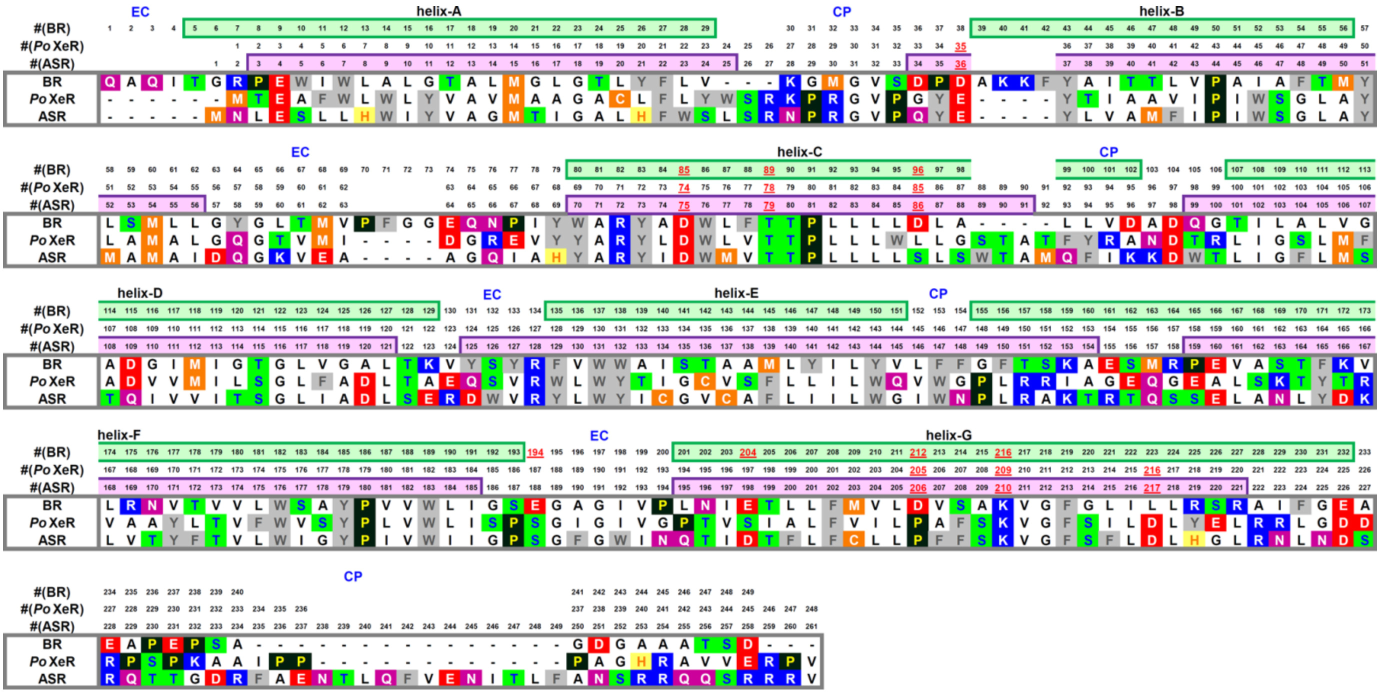

Supplementary Figure 1 Sequence alignment of selected microbial rhodopsins. The alignment of amino acid sequences of BR, *Po*XeR and ASR. The numbers of their motifs, the homologous residues to the secondary counterion of BR (BR D212), the lysine residue forming Schiff-base linkage with retinal and the residues involved in H^+ transport are highlighted as red and underlined characters. The positions of transmembrane helices are indicated by green and purple rectangles for BR and ASR according to their X-ray crystallographic structures (PDB ID: 1M0L (BR) and 1XIO (ASR)), respectively.

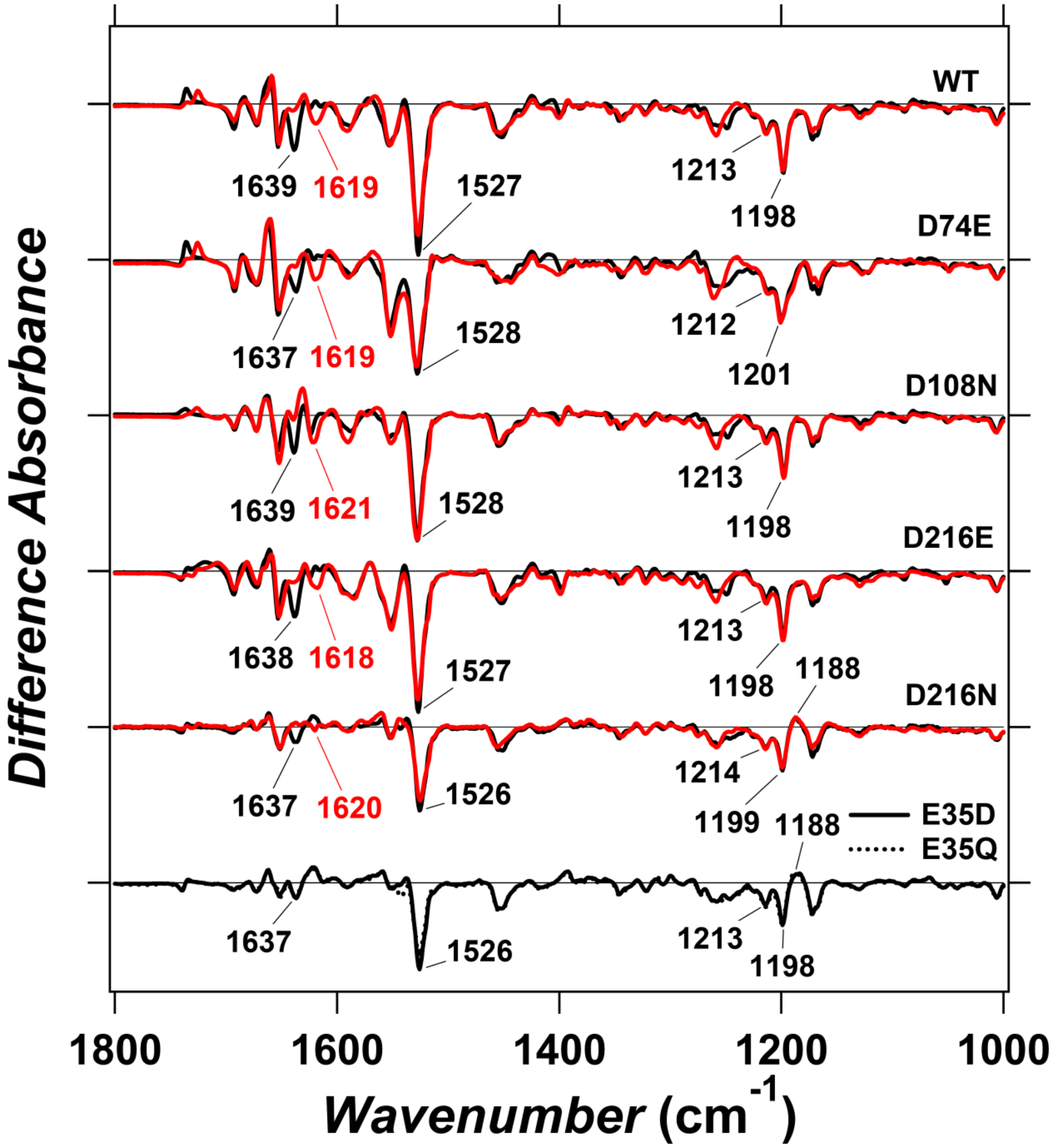

Supplementary Figure 2 Light-induced FTIR difference spectra of *Po*XeR mutants in the 1800-1000 cm^{-1} region at *T* = 230 K and pH 8.0. Black and red lines represent the samples hydrated with H_2O and D_2O, respectively.

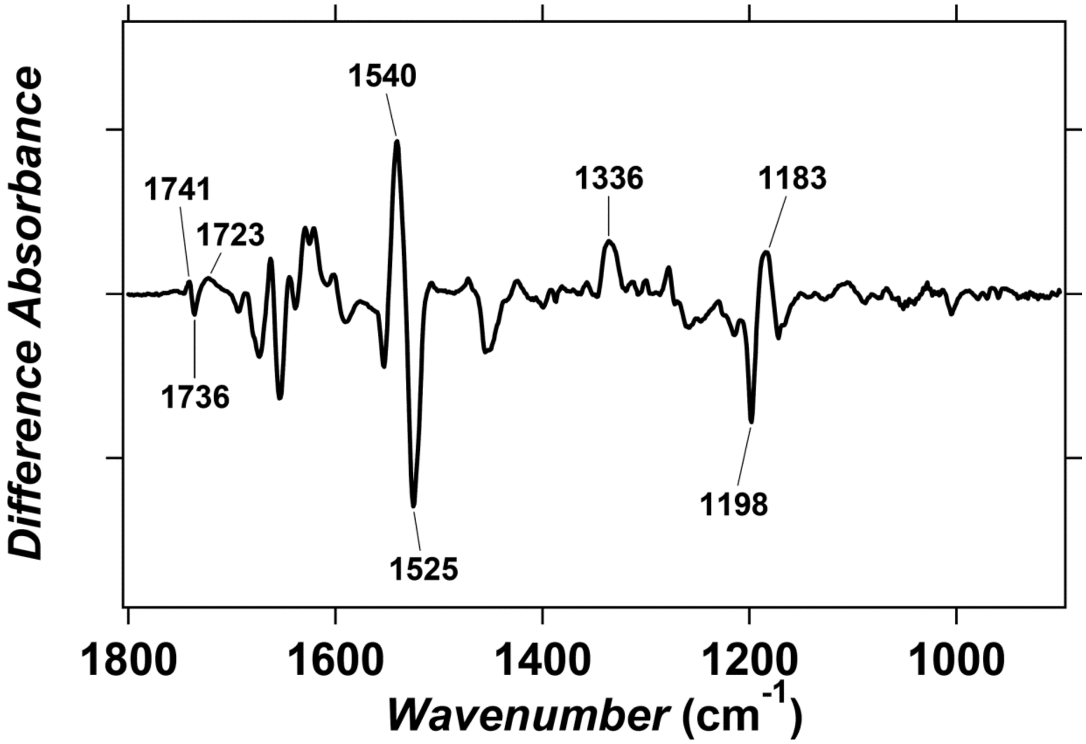

Supplementary Figure 3 Light-induced FTIR difference spectra of *Po*XeR mutants and *Po*XeR$_{13C}$ in the 1800-1000 cm^{-1} region at *T* = 230 K and pH 8.0. The sample was hydrated with H_2O.
